# The Quality Assessment of Stored Red Blood Cells Probed Using Atomic-Force Microscopy

**DOI:** 10.1155/2014/869683

**Published:** 2014-12-28

**Authors:** I. M. Lamzin, R. M. Khayrullin

**Affiliations:** The Department of Human Anatomy, Ulyanovsk State University, Ulyanovsk 432017, Russia

## Abstract

At the moment the suitability of stored red blood cells (sRBC) for transfusion is checked by routine methods such as haemoglobin estimation and the level of haemolysis. These methods cannot characterize directly the quality of the membranes of sRBC. The aim of this work is to assess the quality of sRBC based on such criteria as the membrane's stiffness and the size and the form of sRBC. *Materials and Methods.* We have investigated 5 series of dry cytosmears of the sRBC which had been kept in blood bank in a period from 1 to 35 days. After AFM imaging, in every specimen, 5 RBC were chosen at random; the diameter, the height, and the stiffness were measured on each of them. *Results.* The present study shows high increase of the mean values of YM and height of RBC after 35 days of storage and decrease of the mean values of their diameter. *Conclusion.* Statistically significant high increase of the mean values of YM indicates the decrease of the elasticity of the cells in the course of storing of the RBC. This parameter along with the morphological characteristics can be used as criterion for assessment of applicability of the sRBC for blood transfusion.

## 1. Introduction

Since the atomic-force microscopy has been invented, a great number of researches dedicated to its usage in cytobiology and medicine have been carried out. A large number of the researches show that AFM enables visualizing the biological objects with high definition and measuring their biophysical characteristics; however, it does not provide a well-structured system of assessment criteria and interpretation of the obtained results. This results in a routine application of AFM in medicine. This work includes the composite assessment of the quality of the sRBC probed using AFM.

At the moment the suitability of sRBC for clinical usage is checked by routine methods such as haemoglobin estimation, haematocrit, and the level of haemolysis at the end of the period of validity of the blood components. These tests are able to give solely indirect evidence of the condition of the RBC membranes which have not undergone haemolysis. This fact prevents from making any conclusions on the structural and functional value and, as a result, on the quality of the blood component for transfusion. At the same time RBCs are easily visualized objects with the use of AFM: they are distinctively separated from each other in the cytology samples, have specific form, and retain their structural features [1]. The ability to retain the shape is determined by the developed perimembrane cytoskeleton—a two-dimensional regular-shaped protein net consisted of several types of proteins: spectrin, actin, ankyrin, and so forth [2]. The proteins of the cytoskeleton are closely connected with transmembrane channels and receptors; this defines the dependence between the structural stability of the cell, its form, stiffness, and its functional activity. In course of erythrocytes ageing, the level of intracellular ATP decreases [3]. The energy failure leads to the slowing down of reparative changes of the cytoskeleton structural elements. The erythrocytes steadily lose their biconcave form altering, first, to echinocytes and, then, immediately prior to haemolysis, to spheroechinocytes. All these structural changes are to be followed by the changes in biophysical characteristics of the membranes and the form of the cells.

The aim of this work is to research the changes of RBC membranes stiffness in sRBC and the form and the size of RBC probed using AFM in the course of storage within 35 days at standard temperature conditions and make a conclusion on the application of these transfusion media for clinical usage.

## 2. Materials and Methods

The research has been carried out according to the requirements of the current legislation of the Russian Federation, ethical standards, and principles of the Declaration of Helsinki (1964) with all the following amendments and revisions that regulate biomaterials scientific researches as well as the international ethical guidelines for biomedical research involving human subjects prepared by the Council for International Organizations of Medical Sciences (CIOMS). The research protocol has been approved by the Ethical Committee of Ulyanovsk State University of the Ministry of Education and Science of the Russian Federation. For this research 44 nonrepeated specimens, packed in 450-mL blood bags by “Baxter” (USA) with the use of the additive “CPDA-1,” were chosen randomly from sRBC banked in “Ulyanovsk State Blood Centre.” All samples were divided into 5 study groups. The first grouped dry samples of RBC are made of sRBC at the day of the donation. The second, third, fourth, and fifth series included dry specimens of RBC made of sRBC kept in the blood bank at a temperature 4°C for 7, 14, 21, and 35 days, respectively.

### 2.1. Preparing Samples

The dry specimens were prepared from sRBC which had been stored with the use of the additive. Before the test, sRBC were carefully mixed in the blood bag, the port was opened up, and a blood spot was put on a glass slide and spread evenly by a spreading rod. We used fine polished glasses with a constant thickness of 1,0 mm and 76∗25 mm in area for cytosmears preparation. The sample was air-dried for 20 minutes and then was scanned by AFM.

### 2.2. Atomic-Force Microscopy

The scanning was done with the help of the AFM “SOLVER P47-Pro” (by “NT-MDT” Russia) equipped by titanium cantilever with a radius of tip rounding of 10 nm and a special software “Nova” V1.1.0.1847. The values of the apical radius of the cantilever and the elastic constant were taken from the operational technical manual issued by the producer. A semicontact method with 300 kHz frequency output was applied. The scanned area was 120∗120 *μ*m. On each sample five erythrocytes were chosen arbitrarily; their diameter and height were measured; the stiffness of every RBC was checked in 9 points in accordance with the maximum functionality of the modification of the used AFM. Every time after the process of imaging the cells, a virtual square grid divided into 9 equal zones was applied to the image with the help of the special software. In every case the grid was corresponding with the size of the cell. A cantilever contacted an erythrocyte membrane consecutively in each zone of this virtual grid. 40 erythrocytes were scanned in the study group one. Groups two, three, and four each provided 25 erythrocytes for scanning; group five contains 105 erythrocytes. The number of the measures totaled 1980 (220 cells out of 44 specimens). Plotting of force curves and quantitative assessment of the membranes stiffness were done on the basis of the acquired data by means of the AFM bundled software “Nova.”

### 2.3. Young's Modulus Determination

The quantitative evaluation of the membrane stiffness was done in terms of the YM. The Hertz model was applied to determine the YM based on the force curves [4]. The cantilever action force depending on the depth of the indentation in the substrate was defined by the formula

(1) $$\begin{array}{c} F=\frac{4\sqrt{R}}{3}E*\Delta h^{1,5}, \end{array}$$

where *F* is force applied to the specimen; *R* is radius of the tip rounding; Δ*h* is depth of the indentation in the surface; and *E* is YM. Taking into consideration the radius of the cantilever tip rounding, the formula for YM was determined as follows:

(2) $$\begin{array}{c} E=7,5*10^{-3}*\frac{F}{\Delta h^{1,5}}, \end{array}$$

where *F* is Δ*y* of the force graph and Δ*h* is Δ*x* of the force graph. On the graph made by “Nova,” an area of a linear variation of the force curve value showing the application of the cantilever on the surface of the erythrocyte was chosen for Δ*x* and Δ*y*.

### 2.4. Statistical Analysis

Statistical processing of the data was done with the help of the licensed software “Statistica 8.0” StatSoft Inc. (USA) in accordance with the rules of the parametric statistics suggested by the International Committee of Medical Journals Editors (ICMJE). The distribution of the results obtained was checked by the normality tests. Arithmetic mean, standard deviation, 95% confidence interval (CI), and variation coefficient were calculated; analysis of variance (ANOVA) with calculating an *F*-test and Bonferroni correction was done.

## 3. Results

The AFM images of the dry samples of sRBC showed erythrocytes whose forms varied according to the period of storage. The samples produced on the first day of storage included the erythrocytes of a typical biconcave shape, predominantly discocytes. The mean value of the diameter of the erythrocytes was 9,67 ± 0,55 (M ± *σ*) *μ*m; the mean value of the height was 0,49 ± 0,09 *μ*m. The shape of the main part of erythrocytes of the second group was typical—discocytes. The mean value of the diameter of the erythrocytes was 9,41 ± 0,49 *μ*m; the mean value of the height was 0,50 ± 0,11 *μ*m. The shape of a part of the RBC from the third group of samples was changed; they were echinocytes. The mean value of the diameter of the erythrocytes of the third group was 9,04 ± 0,89 *μ*m; the mean value of the height was 0,52 ± 0,11 *μ*m. A portion of erythrocytes from the fourth group had altered shape; there were echinocytes and spheroechinocytes. The mean value of the diameter of the erythrocytes was 8,87 ± 0,74 *μ*m; the mean value of the height was 0,60 ± 0,11 *μ*m. On the surface of the dry sample of the sRBC after 35 days of storage the shape of the erythrocytes is significantly transformed; echinocytes and spheroechinocytes prevail. The mean value of the diameter of the erythrocytes was 7,59 ± 1,07 *μ*m; the mean value of the height was 0,65 ± 0,13 *μ*m. To complete a quantitative description of the shape alteration, we report the results for the mean values of cell diameter, height, and some statistical data in Table 1.

The value of erythrocytes diameter of all five groups according to ANOVA *F*-test is *F* = 52,18 when *P* < 0,001. The value of Bonferroni test for the fifth group shows the difference in values of the erythrocyte diameter in comparison with the rest of the groups for *P* < 0,0001. The value of the erythrocyte diameter of the fourth group of RBC differs from that of the second group for *P* < 0,016. The result of ANOVA *F*-test of erythrocyte height for the series of samples is *F* = 18,89 when *P* < 0,001. Bonferroni test indicates the difference between the values of the fifth group and those of the first, second, and third groups of samples (*P* > 0,0001). The values of the height of the RBC of the fourth group samples dissimilate from the values of the first group (*P* < 0,0236) and from values of the second group of samples (*P* < 0,0111).

**Figure 1:**
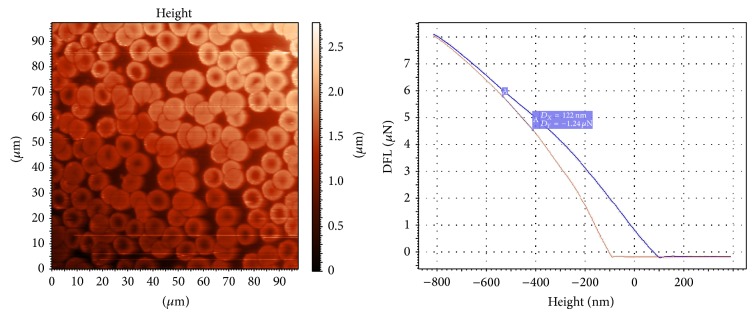
The AFM image of the dry specimen prepared from sRBC after one day of storage and the example of the force curve of the erythrocyte from this sample.

**Figure 2:**
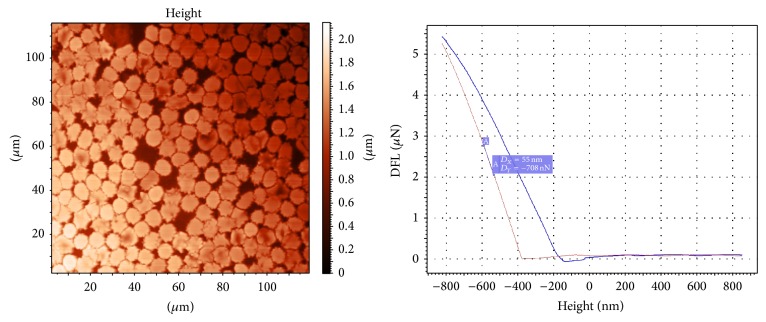
The AFM image of the dry specimen prepared from sRBC after 35 days of storage and the example of the force curve of the erythrocyte from this sample.

The force curves obtained as a result of imaging were used for calculating YM values of the erythrocytes of each group (Figures 1 and 2). The distribution was in accordance with the criterion of normality. The YM mean of the first group was 1,81 ± 0,44 (M ± *σ*) KPa. YM mean value for the fifth group was 3,23 ± 0,70 KPa. YM mean values of five series of sRBC samples and some statistical data are shown in Table 2.

**Figure 3:**
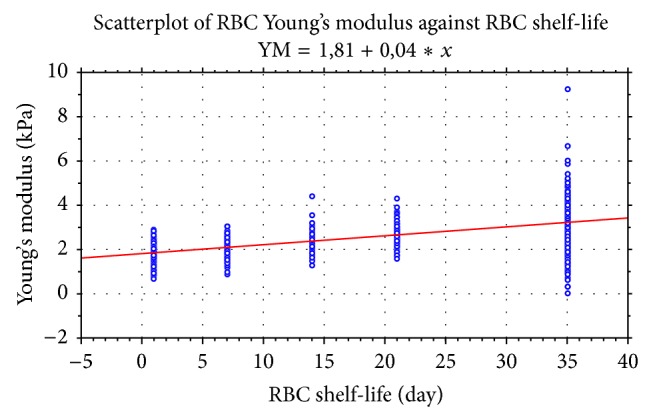
Linear regression of the change of the YM values (estimated with the usage of Hertz model) of the erythrocytes of sRBC in the course of storage.

The result of ANOVA *F*-test of YM for five series of samples is *F* = 442,3 when *P* < 0,001. The change of the YM values of the erythrocytes of sRBC presents the linear regression (Figure 3). The formula of the regression was derived and defined as follows: YM = 1, 81 + 0, 04∗*x*, with *x* being the number of days RBC were stored.

## 4. Discussion

The issue of the composite quality assessment of the blood components produced by blood centers is one of the most burning questions of contemporary transfusiology since nowadays clinical pathways for many diseases and conditions include blood component transfusion as a part of supportive medical care. In accordance with regulatory requirements, the quality assessment of sRBC on the compliance with parameters stated in the technical regulations is done by checking the free residual haemoglobin level, haematocrit, or the hemocytolysis level at the end of the storage period [5]. Nevertheless, the abovementioned routine technologies are not able to characterize the qualities of the red cell ghosts membranes left in sRBC and consequently the genuine qualities of the latter. A modern technology applied for investigating the membranes condition of the biological objects is an atomic-force microscopy [6]. It is based on the principles of nanoscale measurements, used for examining the qualities of membranes of alive or fixed cells, for particularization of the structure of their surfaces and determining their stiffness [1]. So, for example, AFM was applied to visualize a number of deformations of erythrocytes induced by various chemicals [7]. A group of scientists has found out deficiencies of erythrocytes membrane structures while probing the blood by AFM in patients suffering from systemic lupus erythematosus [8]. With the help of AFM it was demonstrated that hypertension and diabetes mellitus correlate with reduction of RBC elasticity [2, 9] and erythrocytes lose capacity to flow in capillaries without hindrance. Membrane stiffness may alter the process of blood preservation, separation, and storage. In the present work, an AFM scanning of erythrocytes was done using intermittent mode which is sensible for studying bioobjects both in ambient air and in liquid medium. This mode enables visualizing large molecules, membrane structures, and its relief without significant distortions and damages of the cell surface [8, 10]. Dry cytosmears of the RBC were prepared in order to assess the quality of erythrocytes since exsiccation does not change their form and membrane structures significantly [11, 12]. Scanning of dry samples provides an opportunity to record minimal distortions of the membrane structure, whereas spatial resolution in liquid medium is to a great extent limited [13]. When choosing 5 erythrocytes on each blood sample, we were not trying to characterize the exact sample using the features of those cells. A large number of samples help to receive the information about the quality of whole array of RBC being stored in a blood bank during the same time period.

The received data correlates with the results of the measurements of the dried RBC stiffness made with the help of similar microscopes models (NT-MDT Company) [14]. This research of the erythrocytes, kept in sRBC, carried out using AFM showed that YM of RBC after 35-day storage has significantly increased in comparison with that of the first day. We have also found out, according to the data received by AFM, that the form of preserved erythrocytes in the course of long-term storage within standard temperature conditions undergoes some changes similar to those detected via optical microscopy. If, on the day of collection, there are biconcave discocytes which prevail, a big number of echinocytes and spheroechinocytes are to be found at the end of the storing period. The degree of changes can be related to destructive processes of membrane and perimembrane cytoskeleton ageing correlated with decrease of ATP reserves, peroxidation, and enzymic oxidation in sRBC, which yet do not lead to haemolysis or do not increase the level of free haemoglobin. In the course of ageing and necrocytosis YM of the membranes changes immensely [15]. Almost two-time augmentation of erythrocytes membrane YM value within a standard storage time indicates the increase of membrane stiffness, so the life cycle of changed erythrocytes has to be much shorter than that of biconcave erythrocytes with low YM values. As judged by the values of the variation coefficient of the biophysical parameters, it is possible to conclude that erythrocyte population, both on the day of the blood preservation and on the last day of storage, maintains the high level of heterogeneity. The heterogeneity seemed to be connected with different “age” of the circulating cells. We have determined a considerable difference between the CI values of the samples from the first (1,76 ÷ 1,86 KPa) and the fifth (3,19 ÷ 3,27 KPa) groups as well as the absence of zone with overlapping values. This fact indicates that processes of ageing of the erythrocytes, circulating actively in a bloodstream, are followed by a different dynamics of biophysical membrane values as compared to those of the cells being stored in artifactual conditions.

## 5. Conclusion

The present work shows that AFM, a modern nanotechnological tool for assessing the local stiffness of cell membranes, can be applied to determine an YM of erythrocytes in sRBC. This research has revealed a statistically significant increase of YM values of RBC impaired and followed by the alteration of their form to echinocytes and spheroechinocytes in the course of long-term storage of sRBC within 35 days at standard temperature condition of +4°C. In summary, YM along with such morphological characteristics as the height and the diameter of the cell can be applied as immediate criteria for assessment of applicability of sRBC for blood transfusion.

## Figures and Tables

**Table 1:** Statistical analysis of erythrocytes diameter and height values of five groups of sRBC samples.

Characteristics	Day	*N*	M ± *σ*	95% CI	CV%
Diameter	1	40	9,67 ± 0,55	9,44 ÷ 9,90	5,7
Height	1	40	0,49 ± 0,09	0,45 ÷ 0,53	19,3
Diameter	7	25	9,41 ± 0,49	9,26 ÷ 9,57	5,2
Height	7	25	0,50 ± 0,11	0,47 ÷ 0,54	21,9
Diameter	14	25	9,04 ± 0,89	8,67 ÷ 9,41	9,9
Height	14	25	0,52 ± 0,11	0,47 ÷ 0,57	21,3
Diameter	21	25	8,87 ± 0,74	8,56 ÷ 9,18	8,4
Height	21	25	0,60 ± 0,11	0,55 ÷ 0,64	19,1
Diameter	35	105	7,59 ± 1,07	7,38 ÷ 7,80	14,1
Height	35	105	0,65 ± 0,13	0,63 ÷ 0,68	19,8

**Table 2:** Statistical analysis of the erythrocytes membrane stiffness (estimated with the usage of Hertz model) of five series of sRBC samples.

Parameter	Day	*N*	M ± *σ*	95% CI	CV%
YM	1	360	1,81 ± 0,44	1,76 ÷ 1,86	24,6
YM	7	225	2,21 ± 0,38	2,15 ÷ 2,26	17,3
YM	14	225	2,34 ± 0,33	2,29 ÷ 2,37	13,9
YM	21	225	2,67 ± 0,39	2,62 ÷ 2,72	14,6
YM	35	945	3,23 ± 0,70	3,19 ÷ 3,27	21,6
